# Precision medicine in rheumatoid arthritis: advances and clinical applications of multi-omics biomarkers

**DOI:** 10.3389/fimmu.2026.1876960

**Published:** 2026-09-18

**Authors:** Desheng Wu, Lin Chang, Juan Chai

**Affiliations:** 1 Department of Orthopedics, Bishan Hospital of Chongqing Medical University, Chongqing, China; 2 Department of Blood Transfusion, Bishan Hospital of Chongqing Medical University, Chongqing, China; 3 Department of Equipment and Supplies, Bishan Hospital of Chongqing Medical University, Chongqing, China

**Keywords:** advances and clinical applications, artificial intelligence, multi-omics biomarkers, precision medicine, rheumatoid arthritis

## Abstract

Rheumatoid arthritis (RA) is a highly heterogeneous autoimmune disease characterized by the presence of synovitis, joint destruction, and systemic inflammation. Despite the substantial improvement in patient outcomes brought about by the advent of targeted therapies, the traditional “one-size-fits-all” treatment model still faces numerous challenges. These include individual variability in treatment response, a “trial-and-error” process in drug selection, and the emergence of refractory RA. Precision medicine is the development of optimal prevention and treatment strategies based on patients’ individual characteristics (including genetic, environmental, and lifestyle factors). This new approach offers a way to address the therapeutic challenges of RA. Multi-omics biomarkers, which integrate data from multiple dimensions including genomics, transcriptomics, proteomics, metabolomics, and epigenomics, are central tools for achieving precision medicine in RA. This article reviews the current status and challenges of precision medicine for RA, systematically summarizing the latest research advances in multi-omics biomarkers regarding RA pathogenesis, early diagnosis, treatment response prediction, prognosis assessment, and risk stratification of complications. The primary focus of this research is the identification of novel biomarkers [including TAOK3 and *S*-adenosylmethionine (SAM)] and the subsequent assessment of their clinical significance. Additionally, the study explores the application of artificial intelligence (AI) technology in the integration of multi-omics data and the development of predictive models. This paper analyzes the challenges faced by multi-omics biomarkers during clinical translation. These challenges include technical barriers, cost-effectiveness, and standardization. Distinct from previous reviews, which have focused broadly on the pathogenesis of RA, this review synthesizes cutting-edge evidence on TAOK3 and SAM as emerging translational biomarkers. It also critically evaluates the nascent integration of spatialomics and liquid biopsy technologies within the “multi-omics + AI” framework. Furthermore, it outlines the prospects for the “clinically integrated multi-omics AI model” in propelling RA treatment into a new era of individualization and precision.

## 1 Introduction

Rheumatoid arthritis (RA) is a prevalent chronic autoimmune disease that affects the musculoskeletal system. Its global prevalence is estimated to range from 0.5% to 1.0% (1). The primary pathological feature of this condition is persistent inflammation of the synovial membrane, which ultimately leads to erosion of cartilage and bone, joint deformity, and loss of function. This has severe impacts on patients’ quality of life and increases the risk of complications such as cardiovascular disease (2, 3). RA exhibits significant clinical heterogeneity, encompassing disease activity, the rate of radiographic progression, treatment response, and long-term prognosis (4, 5) (**Figure 1A**).

In recent decades, therapeutic strategies have evolved from conventional nonsteroidal anti-inflammatory drugs (NSAIDs) and corticosteroids to combination therapies centered on disease-modifying antirheumatic drugs (DMARDs). The advent of biologic DMARDs (bDMARDs) and targeted synthetic DMARDs (tsDMARDs) has significantly advanced the implementation of “treat-to-target” (T2T) strategies, yielding improved clinical outcomes (6–8). Nevertheless, current treatment paradigms remain suboptimal. For instance, clinical remission is achieved in only approximately 50% of patients with first-line methotrexate (MTX) monotherapy, while 30%–40% of individuals exhibit inadequate responses to bDMARDs, necessitating frequent drug switching and increased healthcare costs (9–11). This empirical “trial-and-error” approach not only delays optimal intervention but also imposes substantial economic burdens on patients and healthcare systems (12) (**Figure 1B**).

In order to address these challenges, precision medicine has emerged as a critical imperative in the field of rheumatology. Precision medicine is a term used to describe a healthcare approach that aims to individualize treatment plans for patients by leveraging their unique molecular profiles, which are determined by genetic, environmental, and lifestyle factors (13). This approach involves the classification of patients into distinct subgroups based on their molecular profiles, allowing for the administration of tailored therapeutic interventions. Recent technological breakthroughs in multi-omics (genomics, transcriptomics, proteomics, metabolomics, and epigenomics) coupled with advances in artificial intelligence (AI) and bioinformatics have revolutionized biomarker discovery (13–18). These integrative approaches enable systematic dissection of RA pathophysiology across molecular layers, thereby laying the groundwork for a paradigm shift from “one-size-fits-all” to individualized medicine (19) (**Figure 1C**).

This review comprehensively examines the progress and clinical potential of multi-omics biomarkers in RA precision medicine, focusing on their roles in disease diagnosis, treatment response prediction, and prognosis assessment. The focus of this study is the identification of emerging biomarkers [e.g., TAOK3 and *S*-adenosylmethionine (SAM)] and the discussion of AI-driven strategies for the integration of multi-omics data and the development of predictive models (20). In conclusion, a critical evaluation of the technical, translational, and economic challenges in clinical implementation is provided. The future is envisaged in which the synergy of “clinically integrated multi-omics AI model” will drive personalized RA care into a new era of therapeutic precision (**Figure 1D**).

**Figure 1:**
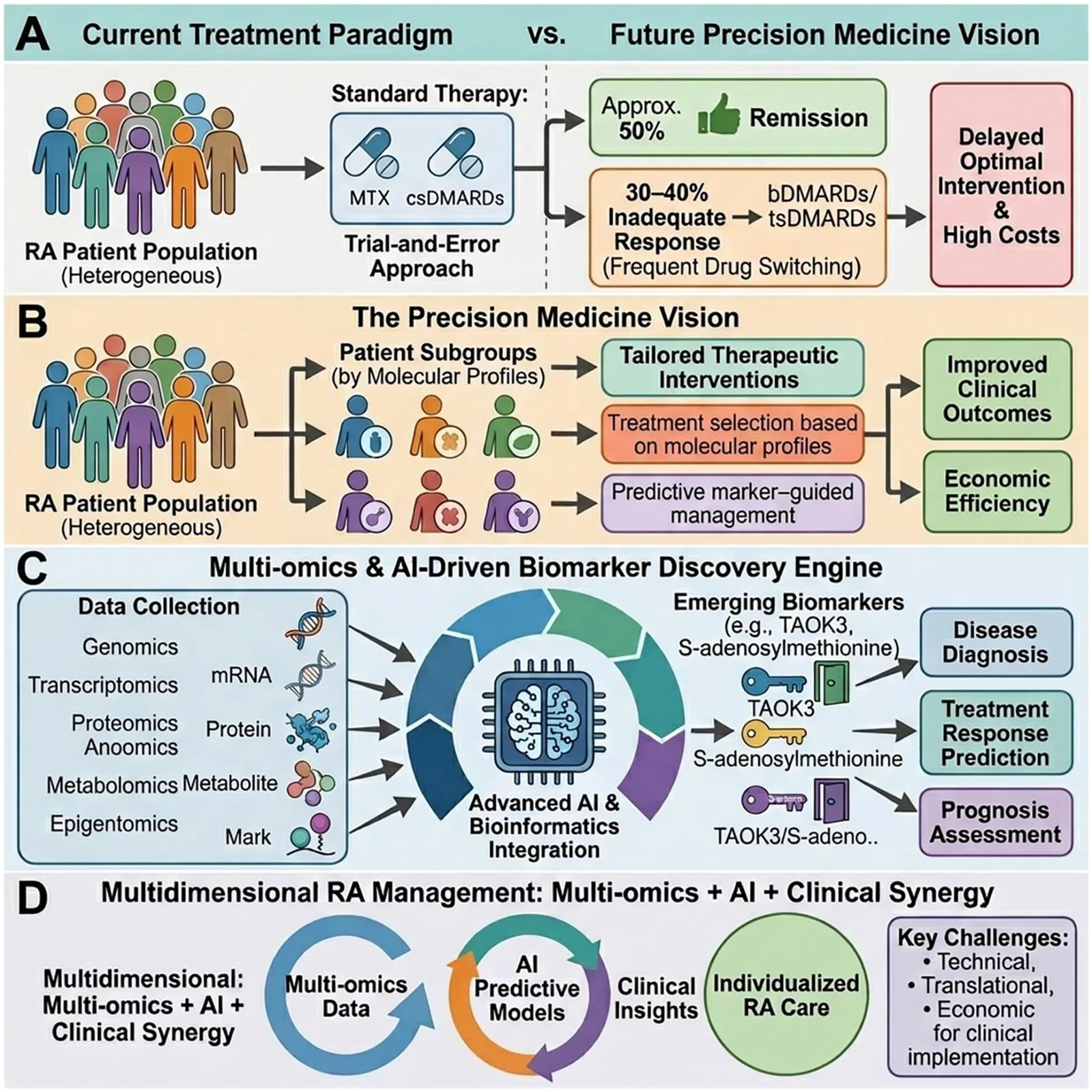
The current dilemma and future blueprint in the field of RA treatment. **(A)** Current treatment paradigm vs. future precision medicine vision. **(B)** The precision medicine vision. **(C)** Multi-omics and AI-drive biomarker discovery engine. **(D)** Multidimensional RA management.

## 2 Status and challenges of precision medicine in RA

### 2.1 Limitations of conventional treatment paradigms

RA treatment is conventionally initiated using a “step-up” or “pyramidal” strategy, whereby therapy is initiated with conventional synthetic DMARDs (csDMARDs), such as MTX (21). In cases where patients demonstrate an inadequate response to csDMARDs, treatment is escalated to combination therapy or switched to bDMARDs {e.g., tumor necrosis factor inhibitors (TNFi) (22)} or tsDMARDs {e.g., Janus kinase inhibitors (JAKi) (23)}. While this approach has been demonstrated to be effective at a population level, it does not take into account the significant interindividual variability that is observed. In clinical practice, physicians frequently resort to empirical “trial-and-error” methodologies, namely, the alteration of medications following the occurrence of therapeutic failure (24). This approach, however, carries the risk of disease progression and irreversible joint damage. Furthermore, even within the same drug class (e.g., different TNFi agents), response heterogeneity persists, suggesting divergent biological mechanisms underlying “intra-class switching” inefficacy (8) (**Figure 2A**).

### 2.2 Preliminary advancements in precision medicine

To date, precision medicine for RA has demonstrated some nascent progress. The most frequently employed biomarkers in clinical practice are rheumatoid factor (RF) and anti-cyclic citrullinated peptide antibodies (ACPAs) (25). ACPA positivity not only is an important criterion for the diagnosis and classification of RA, but also is associated with a more aggressive disease phenotype and a poorer prognosis (26). Some studies suggest that patients with an ACPA-positive diagnosis may demonstrate a heightened response to specific bDMARDs, including abatacept and rituximab (27). Furthermore, the “multi-biomarker disease activity” (MBDA) score (28), based on multiple serum protein markers (such as VCAM-1, IL-6, and TNF-RI), has been used to objectively assess RA disease activity and has shown potential for predicting radiographic progression (29, 30). Targeted therapy itself is also an embodiment of precision medicine; for example, JAKi can specifically inhibit intracellular signaling pathways, offering new options for patients with refractory RA who have an inadequate response to bDMARDs (23) (**Figure 2B**).

Despite the encouraging outlook, the implementation of precision medicine for RA remains encumbered by numerous challenges. Firstly, the predictive efficacy of existing biomarkers remains limited. Despite the diagnostic and prognostic value of RF and ACPA markers, their sensitivity and specificity are inadequate for predicting specific drug responses (31). Secondly, the integration and interpretation of multi-omics data is a highly complex process. The data formats generated by different omics platforms vary significantly, with high noise levels requiring advanced bioinformatics and AI algorithms for multimodal fusion analysis. This, in turn, imposes stringent demands on data processing capabilities and computational resources (32). Thirdly, substantial barriers to clinical translation persist. A plethora of potential biomarkers have been identified in research, yet numerous challenges must be surmounted if these are to be adopted in routine clinical settings. Such challenges include high detection costs, complex procedures, and prolonged timelines. Lastly, the “black box” nature of AI models is a significant limitation with regard to their clinical application (33). Although complex models, such as deep learning, have been demonstrated to exhibit superior predictive performance, their decision-making processes frequently lack transparency and interpretability (34). This has resulted in difficulties for clinicians to fully trust and utilize these predictions for treatment decisions (**Figure 2C**).

**Figure 2:**
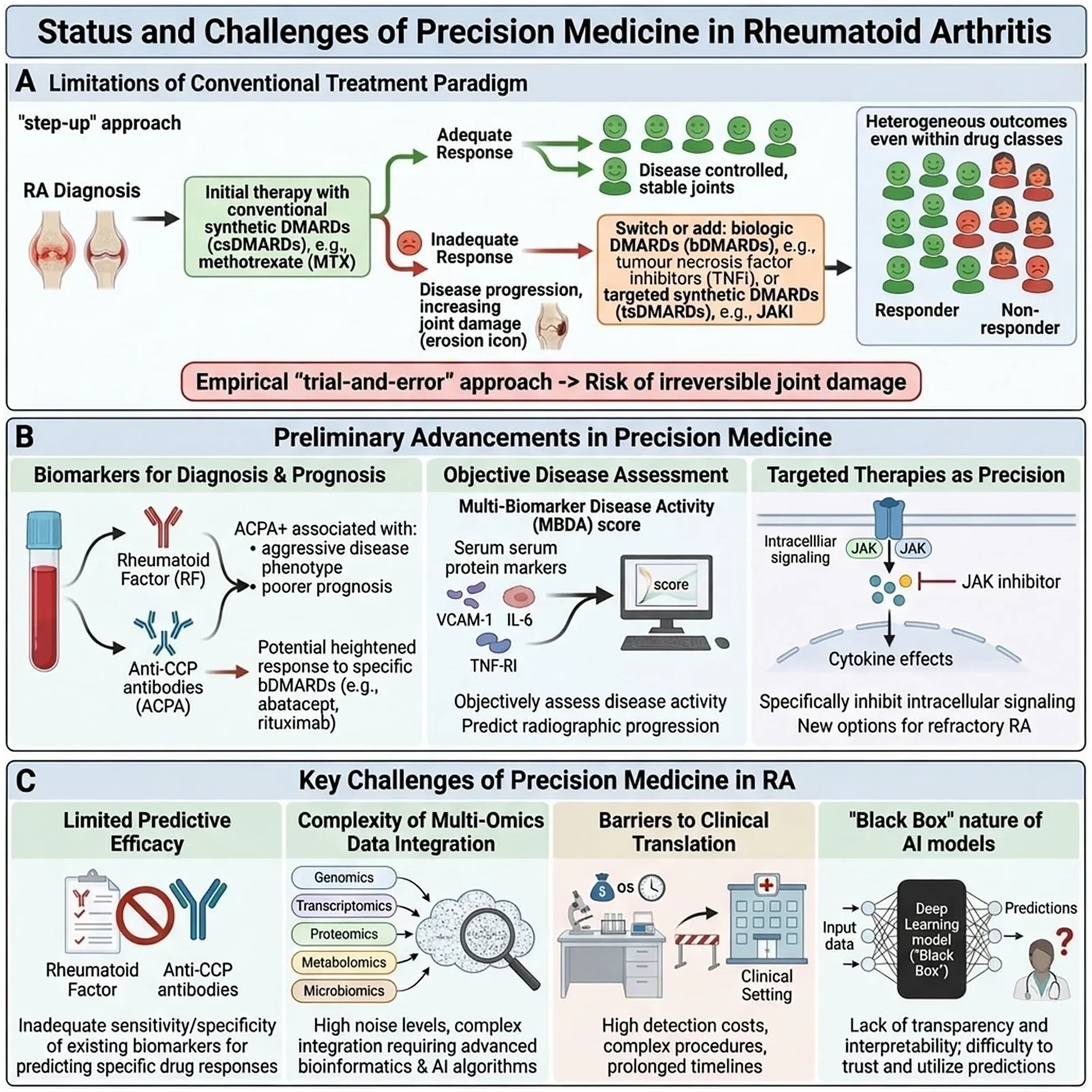
Status and challenges of precision medicine in RA. **(A)** Limitations of conventional treatment paradigm. **(B)** Preliminary advancements in precision medicine. **(C)** Key challenges of precision medicine in RA.

## 3 Advances in multi-omics biomarkers

To overcome these challenges, researchers are leveraging multi-omics technologies to systematically elucidate disease-associated biomarkers across molecular layers in RA pathogenesis, progression, and therapeutic response.

### 3.1 Genomics

Genome-wide association studies (GWAS) have identified over 100 genetic loci linked to RA susceptibility, with the strongest and most consistent signals localized to the human leukocyte antigen (HLA) region (35), particularly the “shared epitope” (SE) alleles in the HLA-DRB1 gene (36). These variants primarily associate with ACPA-positive RA, implicating their role in autoantibody production. Beyond susceptibility genes, pharmacogenomic studies explore genetic determinants of drug response. For example, polymorphisms in the PTPRC gene may influence TNFi efficacy (37). Recently, novel pathogenic mechanisms have emerged, such as the STAT3–NAV2 axis, which regulates synovial hyperplasia and joint destruction, offering new therapeutic targets (38). Although HLA-DRB1 genotyping is well-established within research consortia, its stand-alone clinical utility for individual treatment decisions remains limited due to its low positive predictive value. PTPRC polymorphisms are still in the exploratory stage, awaiting validation in prospective interventional trials (**Figure 3A**).

### 3.2 Transcriptomics

Transcriptomics employs high-throughput sequencing to profile RNA expression dynamics in specific cells/tissues, providing a snapshot of RA pathophysiology. Single-cell RNA sequencing (scRNA-seq) of synovial tissues has unveiled unprecedented cellular heterogeneity in the RA synovium, including distinct subsets of fibroblast-like synoviocytes (FLSs) (39), macrophages (40), and lymphocytes (41). Expanded CD4^+^ T-cell subsets and pro-inflammatory macrophages correlate strongly with disease activity (42). Notably, FLS—the primary effector cells driving joint destruction—exhibit upregulated expression of matrix metalloproteinases (MMPs) (43) and interleukin-6 (IL-6) (44), defining their pathogenic role. Transcriptomic signatures from peripheral blood mononuclear cells (PBMCs) are being developed to predict treatment responses to MTX or bDMARDs (45) **Figure 3B**).

Single-cell multi-omics: A synergistic breakthrough in precision rheumatology. The synergistic application of single-cell multi-omics technologies represents a key modern advancement in the field of precision rheumatology, going beyond traditional batch transcriptomics analysis (46). This technology facilitates the concurrent analysis of multiple molecular levels (e.g., the transcriptome, epigenome, and proteome) within a single cell, which is imperative for a comprehensive comprehension of the substantial heterogeneity of RA. Employing this approach has enabled researchers to redefine the understanding of the synovial microenvironment in RA, leading to the identification of new, functionally distinct subsets of fibroblasts, macrophages, and T cells that were masked in bulk analyses (47). The integration of transcriptomics with other single-cell omics techniques, such as scATAC-seq for chromatin accessibility analysis and CITE-seq for surface protein analysis, has been demonstrated to reveal the gene regulatory networks that underpin specific cellular states. For instance, the activity of a specific transcription factor can be associated with the pro-inflammatory phenotype of a particular FLS subpopulation, thereby facilitating the construction of a more comprehensive pathogenesis map. The identification of cell-type-specific pathogenic pathways is a key objective of single-cell multi-omics, as it facilitates the establishment of more precise therapeutic targets. For instance, interventions can be designed to target pathways that are exclusively active within the pathogenic FLS subpopulation, thereby minimizing the occurrence of off-target effects. In addition, the distinctive molecular traits of these cell subsets can function as highly specific biomarkers, offering valuable insights into predicting treatment response or disease progression (48). These techniques offer tremendous potential for patient stratification in clinical trials or for guiding personalized therapy. For instance, patients exhibiting a high proportion of a particular pro-inflammatory macrophage subset may benefit from treatments that target the key signaling pathways of that subset. In summary, the synergy of single-cell multi-omics is propelling the field from constructing “cell atlases” toward mapping “dynamic functional profiles”, thus laying the foundation for next-generation precision medicine strategies.

### 3.3 Proteomics

Proteomics directly interrogates protein expression, modifications, and interactions—key drivers of biological function. Mass spectrometry (MS)-based approaches have identified dysregulated proteins in RA sera, plasma, and synovial fluid. A groundbreaking study revealed that serine/threonine-protein kinase 3 (TAOK3) is significantly elevated in RA plasma, correlating with DAS28 and Sharp scores (AUC = 0.915 when combined with CRP) (49). Mechanistically, TAOK3 promotes FLS activation and invasion via p38 MAPK signaling. Additionally, mesothelin (MSLN) has emerged as a biomarker of bone erosion, linking epithelial–mesenchymal transition to structural damage (50). Despite the fact that TAOK3 has exhibited remarkable diagnostic performance in this study, it is presently regarded as a candidate biomarker. Further validation is required for the application of the treatment in standard clinical testing. This is due to the fact that its stability across different ethnic populations and its predictive value for treatment response require further validation through multicenter longitudinal cohort studies (**Figure 3C**).

### 3.4 Metabolomics

Metabolomics is the scientific study of small-molecule metabolites, which reflect the ultimate outcome of gene–environment interactions. It provides a unique perspective for understanding the systemic effects of RA. Research has demonstrated that the serum metabolome of patients with RA experiences alterations several years prior to the manifestation of clinical symptoms, characterized by impaired adenosine triphosphate (ATP) production (51), increased oxidative stress (e.g., NAD^+^/glutathione depletion) (52), and elevated levels of pro-inflammatory lipids (e.g., prostaglandins) (17, 53). Dysregulation of the tryptophan–kynurenine (Trp-Kyn) metabolic pathway is considered a key mechanism underlying persistent inflammation in RA (54); metabolite levels in this pathway are closely associated with disease activity and inflammatory cytokine levels. Furthermore, the finding that reduced levels of SAM (55), a key metabolite in the methylation cycle, can serve as a predictor of the progression of bone destruction in patients with RA lends further support to this hypothesis. These metabolic biomarkers not only reveal new pathogenic mechanisms but also offer the potential for developing metabolomics-based tools for early diagnosis and prediction of treatment response. However, many of these metabolomic biomarkers, including SAM, remain in the discovery and validation stages (**Figure 3D**).

### 3.5 Epigenomics

Epigenetic modifications (e.g., DNA methylation, histone marks, and noncoding RNAs) govern gene expression without altering DNA sequences. RA FLS exhibit a stable “aggressive phenotype” driven by aberrant DNA hypomethylation (56), leading to aberrant activation of pro-inflammatory genes (IL-6 and MMP3) (57). As Dai et al. (58) demonstrate, there is a novel modification, namely, histone lactylation (H3K9la), which is coupled to glycolytic metabolism. This modification has been shown to promote M1 polarization in the RA synovium. The enzyme METTL1, which modifies mRNA with m7G, has been shown to regulate the expression of cathepsin B, thereby establishing a link between RNA methylation and FLS activation (59). These findings provide novel insights into the “epigenetic memory” of RA and lay the foundation for the development of epigenetic therapies (**Figure 3E**).

### 3.6 Spatial omics

While the aforementioned omics technologies provide a deep molecular inventory of individual cells or bulk tissue, they often lose the critical architectural context of the tissue microenvironment. Spatial omics technologies address this gap by measuring molecular data (e.g., RNA and proteins) while preserving their two-dimensional or three-dimensional coordinates within a tissue section (60). This is particularly crucial for understanding RA, where the spatial organization of cells within the synovium dictates disease pathogenesis. By mapping the locations of different cell subtypes identified through scRNA-seq, spatial omics can deconvolute the complex synovial microenvironment, revealing specific “cellular neighborhoods” or pathogenic niches where destructive processes are initiated (61). For example, this approach can visualize the co-localization of MMP-producing fibroblasts with immune cells at the pannus–cartilage interface, providing direct evidence of their destructive crosstalk. Furthermore, by analyzing the proximity of different cell types and their ligand–receptor expression profiles, spatial omics can map the *in situ* communication networks that sustain chronic inflammation (62). The spatial organization of immune infiltrates or the expression of specific molecules in defined regions could serve as powerful, spatially resolved biomarkers, paving the way for pathology-informed clinical trials and novel therapeutic strategies that target these pathogenic microenvironments (61) (**Figure 3F**).

**Figure 3:**
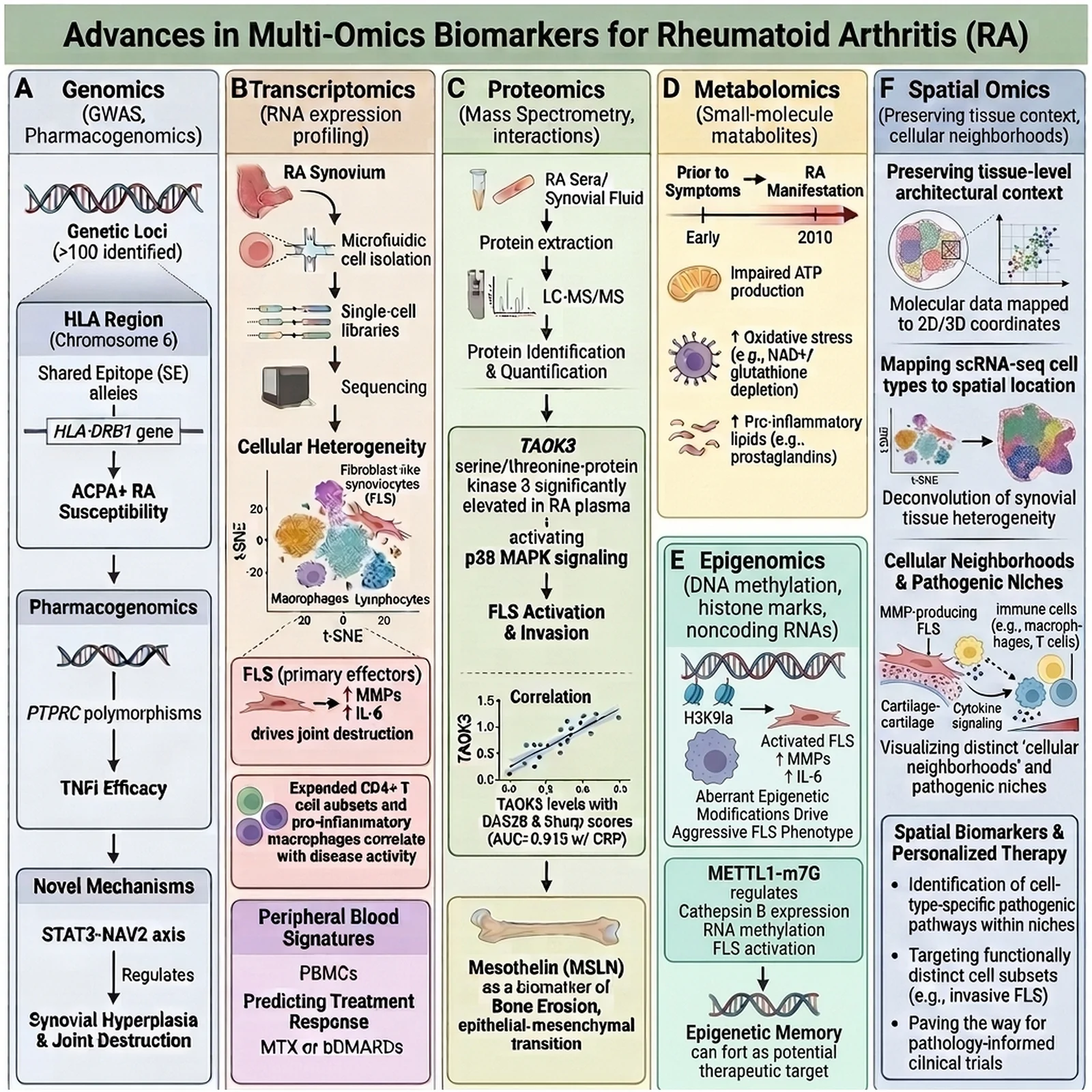
Advances in multi-omics biomarkers for RA. Advances in genomics **(A)**, transcriptomics **(B)**, proteomics **(C)**, metabolomics **(D)**, epigenomics **(E)**, and spatial omics **(F)** for RA.

### 3.7 Current evidence landscape

The process of developing a biomarker from its initial identification in a laboratory setting to its integration into standard clinical practice is a protracted one, necessitating meticulous validation at multiple stages of the process. The current landscape of multi-omics biomarkers in RA is diverse, with different markers positioned at various points along this translational pathway. **Table 1** provides a comprehensive summary of key RA biomarkers, categorized according to their omics layer, level of scientific evidence, and current translational status. This classification system provides a framework for contextualizing the maturity of each biomarker, thereby highlighting the residual gaps that must be addressed to facilitate their clinical implementation.

**Table 1:** Classification of major multi-omics biomarkers in rheumatoid arthritis by evidence level and translational status.

Biomarker/signature	Omics layer	Key findings/mechanism	Level of evidence	Translational status
HLA-DRB1 shared epitope	Genomics	Strongest genetic risk factor for ACPA-positive RA; involved in antigen presentation and breakdown of self-tolerance.	5	Research use (risk stratification); not used for routine treatment decisions.
PTPRC polymorphisms	Genomics	Associated with response to TNF inhibitors in some cohorts, but results are inconsistent.	2	Research use only
Synovial pathotypes (e.g., lympho-myeloid, fibroid)	Transcriptomics	Distinct gene expression signatures in synovial tissue correlate with different cellular infiltrates and predict response to targeted therapies (e.g., rituximab vs. tocilizumab).	3	Investigational use (in clinical trials like R4RA
Peripheral blood IFN signature	Transcriptomics	High interferon gene expression signature in blood predicts non-response to TNF inhibitors.	3	Candidate for validation
Rheumatoid factor (RF)/ACPA	Proteomics (serology)	Autoantibodies central to diagnosis and classification; associated with more severe disease.	5	Routine clinical use
Multi-biomarker disease activity (MBDA) score	Proteomics	A panel of 12 serum proteins (e.g., VCAM-1, IL-6, and CRP) combined into a single score to measure disease activity and predict radiographic progression.	4	Clinical use (limited)
TAOK3	Proteomics	Plasma levels correlate with disease activity and joint damage; promotes FLS activation via p38 MAPK signaling.	2	Research use only
*S*-adenosylmethionine (SAM)	Metabolomics	Reduced serum levels are associated with and predictive of radiographic progression (bone destruction).	2	Research use only
Tryptophan–kynurenine pathway metabolites	Metabolomics	Dysregulated pathway linked to persistent inflammation and disease activity.	2	Research use only
FLS DNA hypomethylation	Epigenomics	Global and gene-specific DNA hypomethylation contributes to the stable aggressive phenotype of RA synovial fibroblasts.	1	Research use only

Level of evidence scale: (1) mechanistic insight (preclinical/basic research); (2) exploratory biomarker (single cohort/cross-sectional); (3) validated biomarker (multiple cohorts/longitudinal); (4) clinical utility demonstrated (prospective/interventional trials); (5) clinical standard. Translational status: research use only; candidate for validation; investigational use; clinical use (limited/routine).

### 3.8 Integration frameworks: from multi-omics data to AI models

The true potency of multi-omics does not lie in the analysis of each data layer in isolation, but rather in the integration of these layers to construct a holistic model of disease (63). However, the integration of heterogeneous, high-dimensional datasets from genomics, proteomics, and metabolomics poses significant computational challenges. In addressing this issue, various machine learning-based integration strategies have been developed, which can be broadly categorized as early, intermediate, and late integration (64) (**Figure 4A**).

Early integration, otherwise known as feature-level concatenation, is the most elementary approach. The process entails the amalgamation of feature vectors from all omics layers into a unified, substantial matrix, which is subsequently introduced into a machine learning model. Despite its apparent simplicity, this method is frequently inadequate due to its inability to accommodate the varying scales and distributions inherent to each data type. It is also noteworthy that the system is susceptible to the so-called “curse of dimensionality” and batch effects from the predominant omics layer (64).

In contrast, late integration, or ensemble modeling, employs a divergent approach. The training of predictive models is conducted separately for each omics dataset. The final prediction is then derived by combining the outputs of these individual models, for instance, through a majority vote or a weighted average. This strategy has been demonstrated to be resilient to missing data in one or more omics layers, and it has been shown to be effective in handling a variety of data types. However, it has the potential to overlook subtle cross-omics interactions that are essential for comprehending the underlying biology (64).

Intermediate integration can be regarded as a powerful compromise, in which each omics layer is first transformed into a common latent space or representation before being combined. This facilitates the modeling of complex relationships both within and between omics layers. Network-based methods are a prominent example of such techniques, with the construction of patient similarity networks or feature interaction graphs being a key feature (65). Advanced techniques like graph convolutional networks (GCNs) can learn from these graph structures to effectively classify patients or identify key biomarkers (66, 67).

A specific and highly effective example of intermediate integration is the multiple kernel learning (MKL) framework, which is often used with support vector machines (SVMs) (68). The clinical challenge of predicting a patient’s response to a TNFi therapy must be considered. The following steps could be taken to construct an MKL-SVM model (**Figure 4B**):

Data input: Collect genomics [single-nucleotide polymorphism (SNP) data], transcriptomics (RNA-seq data), and proteomics (protein abundance data) from a cohort of patients with RA before treatment.

Kernel selection: Assign a specific mathematical kernel function to each omics layer, tailored to its data structure.

1. For genomics data (e.g., thousands of SNPs), a linear kernel is often appropriate. It effectively captures the additive effects of genetic variants.
2. For transcriptomics and proteomics data, which are high-dimensional and may have complex, non-linear relationships with treatment response, a Gaussian radial basis function (RBF) kernel is a superior choice.

Model training: The MKL algorithm then learns an optimal weighted combination of these individual kernels (Kcombined = ΣβmKm), where βm represents the importance of each omics layer “m” in the final prediction. This process simultaneously learns the contribution of each data type while building the classification model.

Despite the efficacy of the resulting MKL-SVM model, it is challenging to ascertain the internal workings of the model, due to the complexity of the algorithm. In order to facilitate the transition to clinical utility, *post-hoc* explanation methods are imperative. SHapley Additive Explanations (SHAP) represents a cutting-edge technique that draws upon the principles of cooperative game theory. It is capable of providing explanations for individual predictions (69). For a given patient, SHAP values quantify the contribution of each feature (e.g., the expression level of TNF-α or the presence of a specific SNP) to the model’s prediction. This facilitates the identification of patients who are likely to be non-responders, as well as the underlying causes, which may include specific pro-inflammatory gene signatures and distinct metabolic profiles. It is imperative that such a high level of interpretability is achieved in order to establish clinical trust and facilitate the transition of these advanced AI models from the research phase to their utilization in clinical settings (70) (**Figure 4C**).

**Figure 4:**
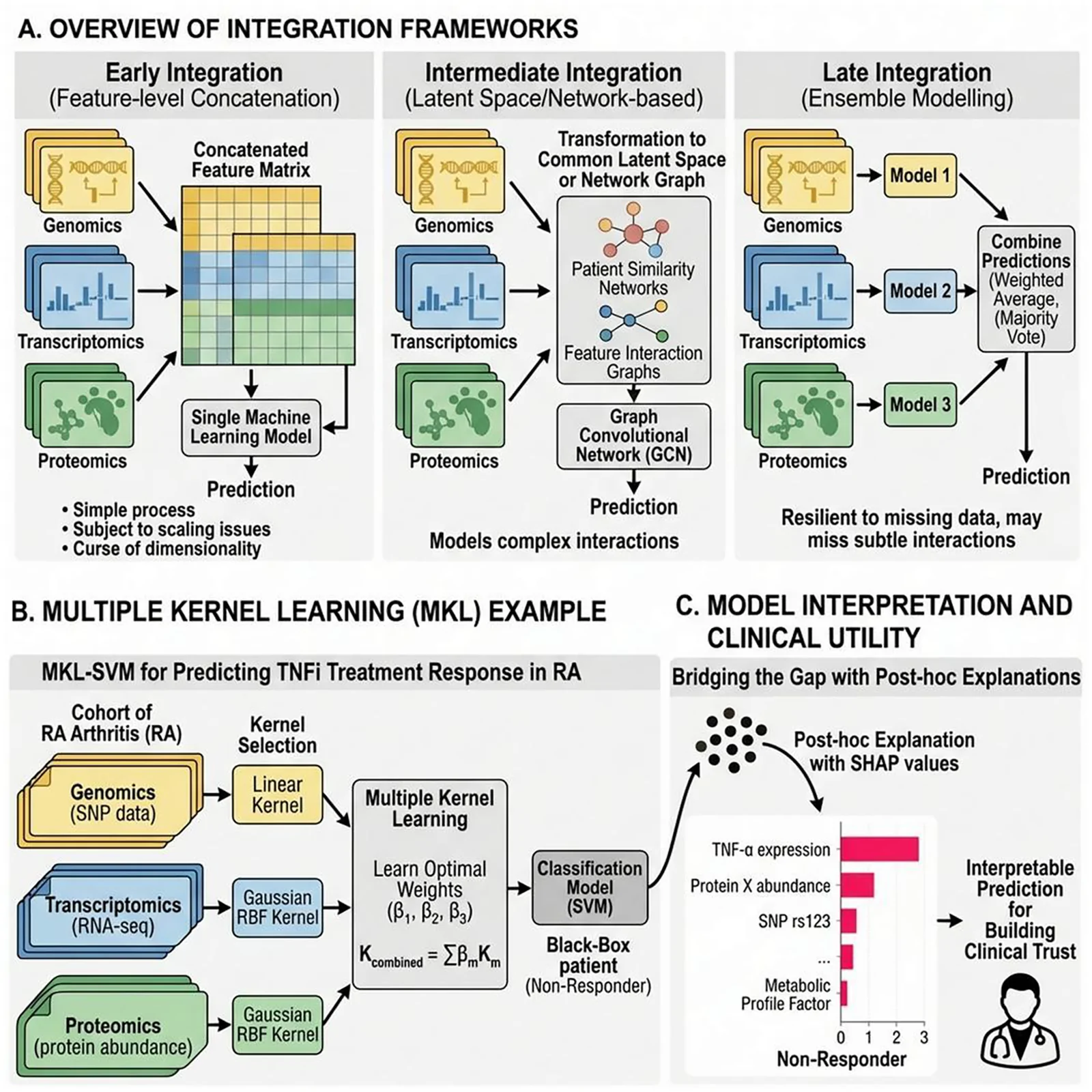
Integration frameworks. **(A)** Overview of integration frameworks. **(B)** Multiple kernel learning (MKL) example. **(C)** Model interpretation and clinical utility.

## 4 The clinical applications of multi-omics biomarkers

The overarching objective of multi-omics technologies is to implement the biomarkers they identify into clinical practice, with the aim of enhancing the patient care process in its entirety (71, 72).

### 4.1 Early diagnosis and risk stratification

The early diagnosis and intervention of RA are of paramount importance in order to prevent joint damage and improve long-term prognosis. However, clinical manifestations are often atypical in the early stages of the disease, posing challenges for diagnosis (73). The combination of multiple biomarker panels has the potential to enhance the accuracy of early diagnosis (74). For instance, the integration of serum metabolic markers (e.g., specific lipid molecules) with imaging markers (e.g., synovial blood flow signals detected by ultrasound) can more sensitively identify high-risk individuals in the “preclinical RA” stage (75). Furthermore, genotyping of populations at risk for arthritis can identify individuals carrying high-risk HLA alleles, thereby enabling targeted monitoring and preventive interventions (**Figure 5A**).

### 4.2 Predicting treatment response

The ability to predict a patient’s response to a specific drug constitutes a fundamental objective of precision medicine for RA (76). The present focus of research is on the development of predictive models based on multi-omics data. For instance, by analyzing the transcriptomic profile of peripheral blood prior to treatment, a gene expression model can be constructed to predict whether a patient will respond to TNF-α inhibitors (77, 78). Furthermore, proteomic biomarkers have demonstrated considerable promise. For instance, as previously referenced, elevated expression levels of TAOK3 have been shown to be indicative of a more favorable response to MTX treatment (7, 49). In a similar vein, metabolomics studies have identified that specific metabolite profiles in pre-treatment serum can differentiate between patients who will and will not respond to csDMARDs therapy. The utilization of these predictive tools will facilitate clinicians in the selection of the most efficacious medications for patients at the commencement of treatment, thereby circumventing the temporal and economic implications associated with ineffective treatments (79) (**Figure 5B**).

### 4.3 Prognosis assessment and disease monitoring

The dynamic monitoring of disease activity and prediction of long-term prognosis are crucial for the adjustment of treatment strategies (80, 81). The advent of multimodal biomarkers has engendered a plethora of novel instruments for this purpose. For instance, serum levels of SAM have been identified as a metabolic biomarker capable of predicting future progression of bone destruction (55). Imagingomics, a term denoting the high-throughput extraction of quantitative features from medical images (e.g., MRI and ultrasound), when combined with AI algorithms, has been demonstrated to offer a more accurate assessment of the extent and changes in synovitis and bone erosion than traditional semi-quantitative scoring systems (e.g., the RAMRIS score for MRI) (82–84). The employment of objective, quantitative metrics facilitates a more precise evaluation of treatment efficacy and facilitates the adjustment of treatment regimens (**Figure 5C**).

### 4.4 Prediction of complications

RA is a systemic disease that frequently manifests with various extra-articular complications. These include RA-associated interstitial lung disease (RA-ILD) and cardiovascular disease, which are the primary causes of mortality in patients (85, 86). The utilization of multi-omics technologies to identify biomarkers capable of predicting the risk of these severe complications is of significant clinical importance. For instance, by analyzing the cytokine profile in peripheral blood or specific autoantibodies (e.g., anti-citrullinated fibrillin antibodies), high-risk patients for RA-ILD can be identified, thus enabling early screening and intervention (87). Conversely, specific metabolic and protein markers associated with lipid metabolism and endothelial dysfunction may assist in identifying subgroups of patients with RA who are at elevated risk of cardiovascular events (17, 88) (**Figure 5D**).

**Figure 5:**
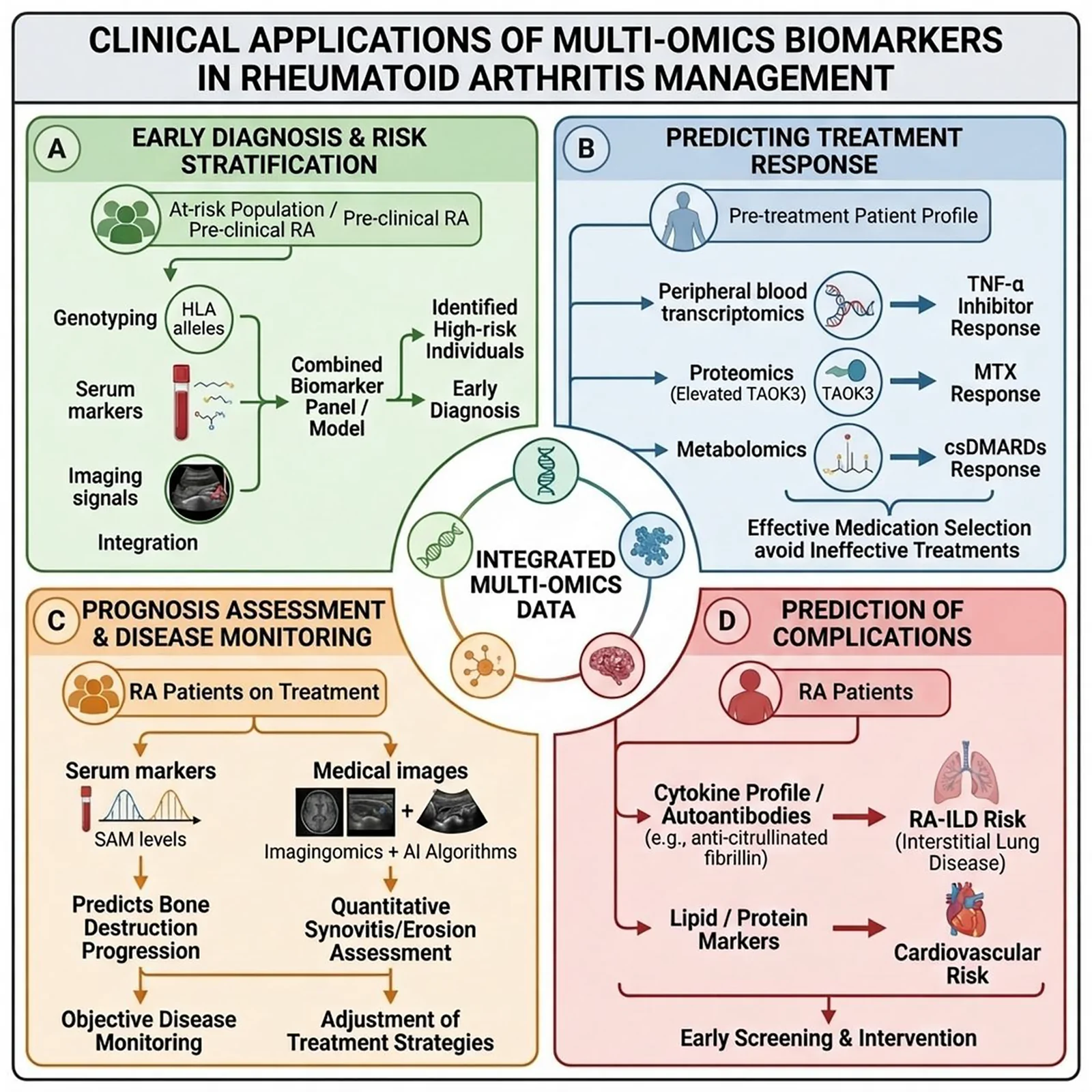
The clinical applications of multi-omics biomarkers in RA management. **(A)** Early diagnosis and risk stratification. **(B)** Predicting treatment response. **(C)** Prognosis assessment and disease monitoring. **(D)** Prediction of complications.

## 5 Challenges and future directions

Notwithstanding the demonstration of significant potential in the field of precision medicine for RA by multiple biomarkers, there remains a considerable distance to travel before laboratory research can be translated into routine clinical practice (25, 80).

### 5.1 Technical and standardization challenges

As has been noted in previous reviews, the potential of MBDA scores and HLA typing has been identified. However, the current bottleneck has shifted towards the interpretability of multi-modal AI models. The primary challenge confronting multi-omics research pertains to the reproducibility and standardization of techniques (89). Data generated by disparate laboratories and technical platforms (e.g., various mass spectrometers or sequencers) frequently exhibit batch effects, which hinders direct comparison and integration. It is therefore vital to establish standardized workflows for sample collection, processing, data generation, and analysis. Moreover, the effective integration of heterogeneous data from diverse omics levels to construct a multimodal AI model capable of comprehensively reflecting disease states represents a significant technical challenge in the field of bioinformatics. This necessitates the creation of novel algorithms that are capable of managing high-dimensional, sparse data and incorporating prior biological knowledge to enhance the robustness and interpretability of the models (**Figure 6A**).

### 5.2 Challenges in clinical translation

The clinical translation of biomarkers necessitates validation through rigorous, multicenter, prospective clinical trials to demonstrate their clinical efficacy and utility (13, 90). The cost-effectiveness of a new biomarker is a pivotal factor in determining its potential for widespread adoption. At present, a significant number of multi-omics assays (for example, proteomics and whole-genome sequencing) continue to be excessively costly, thereby restricting their accessibility in healthcare settings that are constrained by limited resources (91). Consequently, future research must focus on developing more cost-effective and user-friendly diagnostic methods, such as converting complex biomarker panels into enzyme-linked immunosorbent assay (ELISA) or polymerase chain reaction (PCR) assays that can be performed in standard clinical laboratories. Concurrently, rigorous health economic evaluations are required to demonstrate that the long-term health benefits derived from using these biomarkers to guide treatment can offset their upfront testing costs (1) (**Figure 6B**).

### 5.3 Future research directions

Future research will progress towards the development of more minimally invasive, dynamic, and intelligent approaches. Liquid biopsy techniques, including the detection of circulating free DNA (cfDNA), exosome, and circulating tumor cells (CTCs) in plasma, offer the potential for non-invasive monitoring of disease activity and molecular changes in RA (92, 93). For instance, the analysis of microRNAs or proteins in exosome samples derived from synovial cells may offer a more precise reflection of the local pathological state of the joint than the detection of markers in the systemic circulation.

Another pivotal area of focus pertains to the development of AI-assisted real-time dynamic prediction models (20). The integration of patients’ electronic health records (EHRs), multi-omics data, radiomic features, and physiological data collected via wearable devices enables the construction of a dynamic learning system capable of predicting the risk of disease recurrence or changes in treatment response in real time. This system is able to realize the principles of “preventive, participatory, predictive, and personalized” (4P) medicine (94, 95). Furthermore, spatial-omics technology, which facilitates the concurrent acquisition of multiple omics data *in situ* within tissues, will facilitate enhanced comprehension of intercellular interactions and functional dysregulation within the RA synovial microenvironment in the context of tissue architecture, thereby providing more precise spatial information for the identification of novel therapeutic targets (83) (**Figure 6C**).

**Figure 6:**
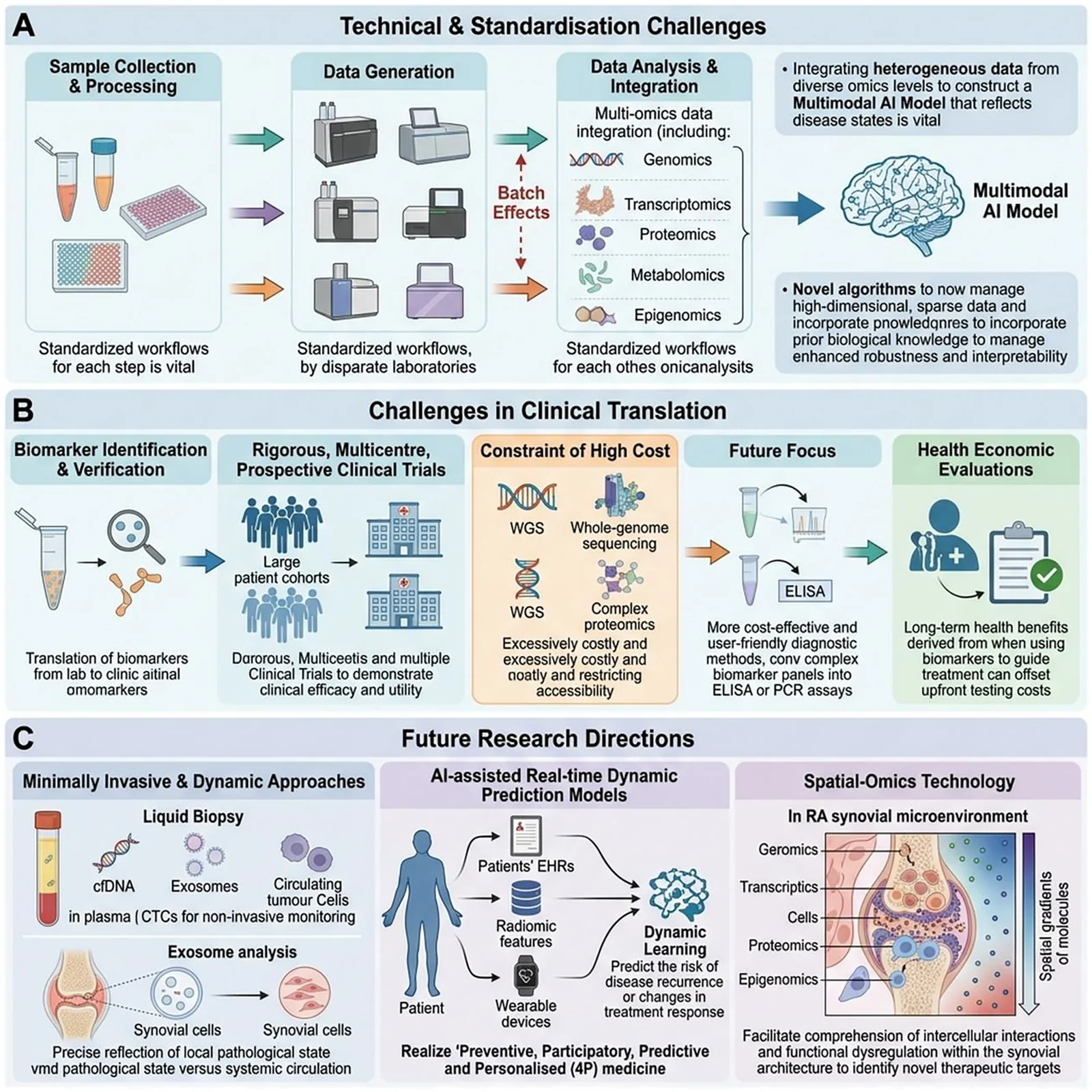
Challenges and future directions. **(A)** Technical and standardization challenges. **(B)** Challenges in clinical translation. **(C)** Future research directions.

## 6 Conclusion

Precision medicine for RA is gradually evolving from a distant concept into clinical reality. Advances in multi-omics technologies provide powerful tools for systematically understanding the heterogeneity of RA and for identifying and validating novel biomarkers (13, 79). From the genome to the metabolome, biomarkers at various molecular levels demonstrate immense potential for application in the early diagnosis, treatment selection, prognosis assessment, and prevention of complications in RA. Nevertheless, the successful translation of these findings into clinical tools that benefit a wide range of patients still requires the overcoming of multiple challenges, including technical standardization, clinical validation, and cost-effectiveness (72, 96).

In the future, a decision-support system that integrates multi-omics data, clinical information, imaging features, and AI algorithms will represent the ultimate form of precision medicine for RA. Achieving this objective necessitates a close collaboration across multiple disciplines, including rheumatologists, biologists, data scientists, and health economists. It is reasonable to hypothesize that the treatment of RA will ultimately move beyond the era of “trial and error” and enter a new era characterized by greater precision, efficiency, and personalization, delivering the best possible health outcomes for every patient.
